# Variation in the Efficacy of Anti-Ulcerative Colitis Treatments Reveals the Conflict Between Precipitating Compatibility of Traditional Chinese Medicine and Modern Technology: A Case of Scutellaria-Coptis

**DOI:** 10.3389/fphar.2022.819851

**Published:** 2022-04-20

**Authors:** Dan Liu, Ran Zhao, Yajing Wu, Yunhong Wang, Rongping Yang, Xiumei Ke

**Affiliations:** ^1^ Chongqing Key Laboratory of Chinese Medicine New Drug Screening, Southwest University, Chongqing, China; ^2^ Chongqing Academy of Chinese Materia Medica, Chongqing, China; ^3^ Chongqing Key Laboratory of Traditional Chinese Medicine for Prevention and Cure of Metabolic Diseases, Chongqing Medical University, Chongqing, China; ^4^ School of Basic Medical Sciences, Jiujiang University, Jiujiang, China

**Keywords:** scutellariae, coptidis, precipitating compatibility, ulcerative colitis, intestinal flora

## Abstract

Scutellariae and Coptidis compose a classical drug pair applied in clinical practice to dispel heat, dryness, and dampness, and they are also precipitation compatible drug pairs. With modern technology, Scutellaria-Coptis is mostly prepared by decocting its components separately, while in the traditional method, it is predominantly prepared as a combined decoction. The present study investigated the effects and mechanisms of separate and combined application of Scutellaria-Coptis decoction on ulcerative colitis (UC) in mice induced by the administration of dextran sulfate sodium (DSS). Changes in body weight, colon length, and Disease Activity Index scores were also evaluated. Hematoxylin and eosin staining and other methods were used to evaluate the overall condition of animals in each group. Intestinal microflora was analyzed using 16S rRNA sequencing, while colon inflammation and antioxidant capacity were evaluated based on the levels of interleukin-6 (IL-6), IL-10, IL-1β, tumor necrosis factor-α, superoxide dismutase, malondialdehyde, and reduced glutathione. The results revealed that Scutellaria-Coptis significantly relieved colon inflammation in mice, and the combined decoction of Scutellaria-Coptis exerted a significant effect on UC. Notably, the protective effect of Scutellaria-Coptis against colon inflammation was weakened when the antibiotic mixture was partially consumed by the gut microbiota. The results of 16S rRNA sequencing showed that the group treated with combined decoction of Scutellaria-Coptis exhibited a higher intestinal microbial diversity and intestinal flora composition than the separated decoction group. Treatment of mice with UC by administering Scutellaria-Coptis decoction through intestinal flora removal (ABX) and fecal microbial transplantation (FMT) was closely associated with intestinal flora composition. In conclusion, Scutellaria-Coptis can relieve UC with an excellent effect especially when taken as a combined decoction, alleviating colon inflammation incurred by intestinal microbes to a certain extent.

## 1 Introduction

Ulcerative colitis (UC) is an acute colonic inflammatory reaction involving the mucosa and submucosa of rectum and colon, with abdominal pain, diarrhea, mucous blood stool, and tenesmus as the primary clinical manifestations. Moreover, UC is a disease that is highly associated with colorectal cancer (Li et al., 2015a; Abdelmegid et al., 2019). The pathogenesis of UC is generally believed to be associated with immune abnormalities, genetic susceptibility to UC, environmental factors, and intestinal flora imbalances (Zhou et al., 2019). Clinical treatment drugs mainly include aminosalicylic acids, glucocorticoids, and immune suppressants (Yu et al., 2017). Long-term use of the drugs results to serious adverse reactions such as the induction of dilated cardiomyopathy and severe heart failure. UC belongs to the “Dysentery,” “Chrysanthemum,” and “Bowel” categories based on the traditional Chinese medicine classification system (Guan et al., 2017)**.** Based on the traditional Chinese medicine (TCM) theory, UC consists of several types of TCM syndromes, the most common of which (34.8%) being damp-heat accumulation syndrome (DHAS). Traditional Chinese medicine has been widely used in the treatment of ulcerative colitis in China, however, it remains challenging to systematically determine its efficacy in the treatment of ulcerative colitis (Guan et al., 2017; Tian et al., 2020).

In the theory of traditional Chinese medicine, both Radix Scutellariae and Rhizoma Coptidis can clear heat, dryness and dampness, purge fire, and detoxify (Chen et al., 2017; Peng et al., 2019). Moreover, a combination of the two herbal components can considerably clear heat, dryness, and dampness. Therefore, both herbs form a classical drug pair for clearing heat, dryness, and dampness in clinical practice (Chan et al., 2020; Luo et al., 2021). According to traditional Chinese medicine practice, Radix Scutellariae and Rhizoma Coptidis decoction is mostly prepared in the form of a combined decoction (Chen et al., 2021). However, in modern industry, the complex reaction between flavonoids, and alkaloids in Radix Scutellariae and Rhizoma Coptidis decoction produces a large amount of precipitate, which is ineffective and destroys the active components, in turn, affecting its clinical efficacy (Chen et al., 2021). The components are often treated separately to avoid the formation of a precipitate during the decoction process. Separated and combined decoctions of Scutellaria-Coptis are the conflicts of traditional precipitation compatibility in drug extraction technology. The clinical efficacy of the Scutellaria-Coptis decoction depends on whether the components are decocted separately or in combination. Presently, a systematic comparison of the therapeutic effects of Scutellaria-Coptis decoction, separately and in combination, on the treatment of UC has not been carried out.

In this study, UC was induced in mice by administering dextran sulfate sodium (DSS). To determine the curative effect of Scutellaria-Coptis decocted separately and in combination, Disease Activity Index (DAI) scores, histopathological inflammatory factors, and antioxidant capacity were evaluated, in addition to the combined effects of Scutellaria-Coptis decoction (Miyazaki et al., 2021). Furthermore, 16S rRNA sequencing was used to analyze the structure and diversity of intestinal flora, and the effect of Scutellaria-Coptis decoction on the intestinal flora of DSS-induced UC in mice was investigated (Li et al., 2015; Liu et al., 2020; Singh et al., 2019; Zhang et al., 2020). The effect of Scutellaria-Coptis decoction on UC and its correlation with intestinal flora were determined using a pseudo-sterile mouse model and a flora transplantation test (Armstrong et al., 2019; Liu et al., 2020; Liu et al., 2021). The scientific extraction technology of Scutellaria-Coptis extract employed in the present study was preliminarily described, providing reference for its clinical use and scientific basis for the preparation process design of Proprietary Chinese Medicine.

## 2 Materials and Methods

### 2.1 Animals

Six- to eight-week-old BALB/c mice (20 ± 2 g) were purchased from Huafukang Biotechnology Company (Beijing, China). The specific pathogen-free animals were raised at the Experimental Animal Center of the Southwest University, Chongqing, China, and the indoor light and dark cycles were maintained at 12 and 12 h, respectively. Relevant experiments were carried out after 1 week of adaptive feeding. All animal experiments were reviewed and approved by the Ethics Committee of the Southwest University, Chongqing, China (Approval Number: YXY202109).

### 2.2 Drugs and Reagents

Radix Scutellariae (Radix) of *Scutellaria baicalensis* Georgi. (Lamiaceae) and Rhizoma Coptidis of *Coptis chinensis* Franch. (Ranunculaceae) rhizome decoction pieces were purchased from Sichuan Xinhehua Traditional Chinese Medicine Co., Ltd. (Sichuan, China). Dextran sodium sulfate (DSS; molecular weight [MW]: 36,000–50,000) was purchased from Dalian Meilun Biotechnology Co., Ltd. (Liaoning, China). Mouse interleukin-6 (IL-6, 20210801–20188a), interleukin-10 (IL-10, 20210801–20162A), interleukin-1β (IL-1β, 20210801–20174A), and tumor necrosis factor-a (TNF-α, 20210801–20852A) ELISA kits were purchased from Enzyme-linked Biotechnology Co., Ltd. (Shanghai, China). Reduced glutathione (GSH), malondialdehyde (MDA), and superoxide dismutase (SOD) detection kits for mice were purchased from Jiancheng Bioengineering Institute Co., Ltd. (Nanjing, China). BCA protein assay kit (batch no. B1513001200) was purchased from Dingguochangsheng Biotechnology Co., Ltd. (Beijing, China). Fecal occult blood test kit (batch no. J18GR152104) was purchased from Yuanye Biotechnology Co., Ltd. (Shanghai, China).

### 2.3 Preparation of Liquid Medicine

The ratio of extract to weight used in the compatibility of Radix Scutellariae and Rhizoma Coptidis pieces in most books and literatures is 1:1.

#### 2.3.1 Decoction Solution

The same amounts of Radix Scutellariae and Rhizoma Coptidis decoction pieces was measured and then fried separately. The decoction pieces were soaked 10 times in pure water for 0.5 h, then fried twice for 1 h each time, filtered, concentrated, and freeze-dried. The freeze-dried powder of Radix Scutellariae and Rhizoma Coptidis was suspended in 0.5% carboxymethyl cellulose (CMC) solution at a concentration of 0.9 g/ml (based on the raw drug volume).

#### 2.3.2 Combined Decoction Solution

Similar amounts of Radix Scutellariae and Rhizoma Coptidis decoction pieces were decocted together. Thereafter, the decoction pieces were soaked 10 times in pure water for 0.5 h, decocted twice for 1 h each time, filtered, combined with a secondary filtrate, which was then concentrated and freeze-dried. The freeze-dried powder of the combined decoction was suspended in 0.5% CMC solution at a concentration of 0.9 g/ml (measured based on the raw drug volume).

### 2.4 Animal Grouping and Drug Administration

#### 2.4.1 DSS-Induced UC in Mice

BALB/c mice were randomly divided into four groups (*n* = 10 per group) after adaptive feeding for 7 days, as follows: 1) Normal Control group (NC), 2) Model group (M), 3) Separated Scutellaria-Coptis decoction group (SD), and 4) Combined Scutellaria-Coptis decoction group (Decocted together, DT). During the experiment, the NC group was administered with distilled water, while the other experimental groups were administered with 3.5% (W/V) DSS solution in drinking water for 10 consecutive days. Moreover, the newly prepared DSS solution was replaced every morning. From the third day of modeling, the treatment groups were administered with 9 g/kg (SD) and 9 g/kg (DT) through regular intragastric administration. The NC and M groups were administered with the same amount of 0.5% CMC solution by gavage once a day, for seven consecutive days.

#### 2.4.2 Pseudo-Sterile Model Group

BALB/c mice were randomly divided into two groups (*n* = 10 per group), as follows: Antibiotic (DT) (ABX [DT]) and Antibiotic (DSS) (ABX [DSS]) groups. Subsequently, 20 male BALB/c mice were intragastrically administered with antibiotic mixture (vancomycin at a dose of 100 mg/kg, neomycin sulfate at a dose of 200 mg/kg, metronidazole at a dose of 200 mg/kg, and ampicillin at a dose of 200 mg/kg) for 5 days to support growth of intestinal flora. Mice were given 2% (W/V) DSS solution in drinking water, and the newly prepared DSS solution was replaced every morning. The DT group was administered with 9 g/kg for seven consecutive days from the third day of modeling.

#### 2.4.3 Fecal Microbial Transplantation Group

BALB/c mice were randomly divided into two groups (*n* = 10 per group), as follows: Fecal microbial transplantation (DT) (FMT [DT]) and Fecal microbial transplantation (DSS) (FMT [DSS]) groups. Afterward, 20 male BALB/c mice were intragastrically administered with antibiotic mixture (vancomycin at a dose of 100 mg/kg, neomycin sulfate at a dose of 200 mg/kg, metronidazole at a dose of 200 mg/kg, and ampicillin at a dose of 200 mg/kg) for 5 days to partially eliminate intestinal flora. After 3 days of intestinal rest, feces from the M and DT groups were re-suspended in phosphate-buffered saline at a concentration of 0.125 g/ml and then 0.15 ml of the suspension was administered to the mice by gavage once a day for 5 days. Thereafter, the mice were given 2% (W/V) DSS solution in drinking water, and the newly prepared DSS solution was replaced every morning. The DT treatment group was administered with 9 g/kg for seven consecutive days.

### 2.5 Disease Activity Index Scores

Mice activity, mental state, diet and drinking water, hair color, and other conditions were observed daily, and weight changes, stool characteristics, and fecal occult blood of each group of mice (detected using the o-toluidine method) were recorded. In addition, the percentage of weight loss, stool characteristics and stool Occult blood were scored using DAI (Chassaing et al., 2014; Liu F. et al., 2020).

DAI = (weight fraction + blood stool trait score points)/3 

### 2.6 Histopathological Analysis

Colon tissue sections (1 cm thick) were cut from the proximal rectal end of the colon, fixed in 4% paraformaldehyde, dehydrated, and conventionally embedded in paraffin blocks (Chassaing et al., 2014; Liu YJ. et al., 2020). Hematoxylin and eosin (H&E) staining was performed and the pathological changes of the colon were observed under a microscope (BA210Digital, Mc Audi Industrial Group Co., Ltd.).

### 2.7 Detection of Inflammatory Markers and Antioxidant Indices in Colon Tissues

An appropriate amount of colon tissues was weighed, ground, centrifuged at 3,000 rpm for 10 min, and the colon tissue homogenates were collected. The contents of cytokines, IL-10, IL-1β, IL-6, and TNF-α in colon tissues were determined according to the instructions of the ELISA kit. In addition, colon tissue homogenates collected from each group were used to determine the contents of MDA, GSH, and SOD according to their respective manufacturers’ instructions.

### 2.8 Fecal Genomic DNA Extraction and 16S rRNA Sequencing

Fecal samples were collected on the third day after administration of treatments. Six mice were randomly selected from each group, and their fresh fecal material collected on a sterile operating table and placed in sterile centrifuge tubes. The collected fecal samples were stored in a refrigerator at –80°C and then sent to Majorbio Bio-Pharm Technology Co., Ltd. (Shanghai, China) for 16S rRNA sequencing.

### 2.9 Statistical Analysis

Statistical analyses were carried out using IBM SPSS Statistics 22.0 (IBM Corp., Armonk, NY, United States), and data were expressed as the mean ± standard (mean ± SD). The differences among groups were compared using analysis of variance.

## 3 Results

### 3.1 A Comparison of the Curative Effect of Separated and Combined Scutellaria-Coptis Decoction on UC

**FIGURE 1:**
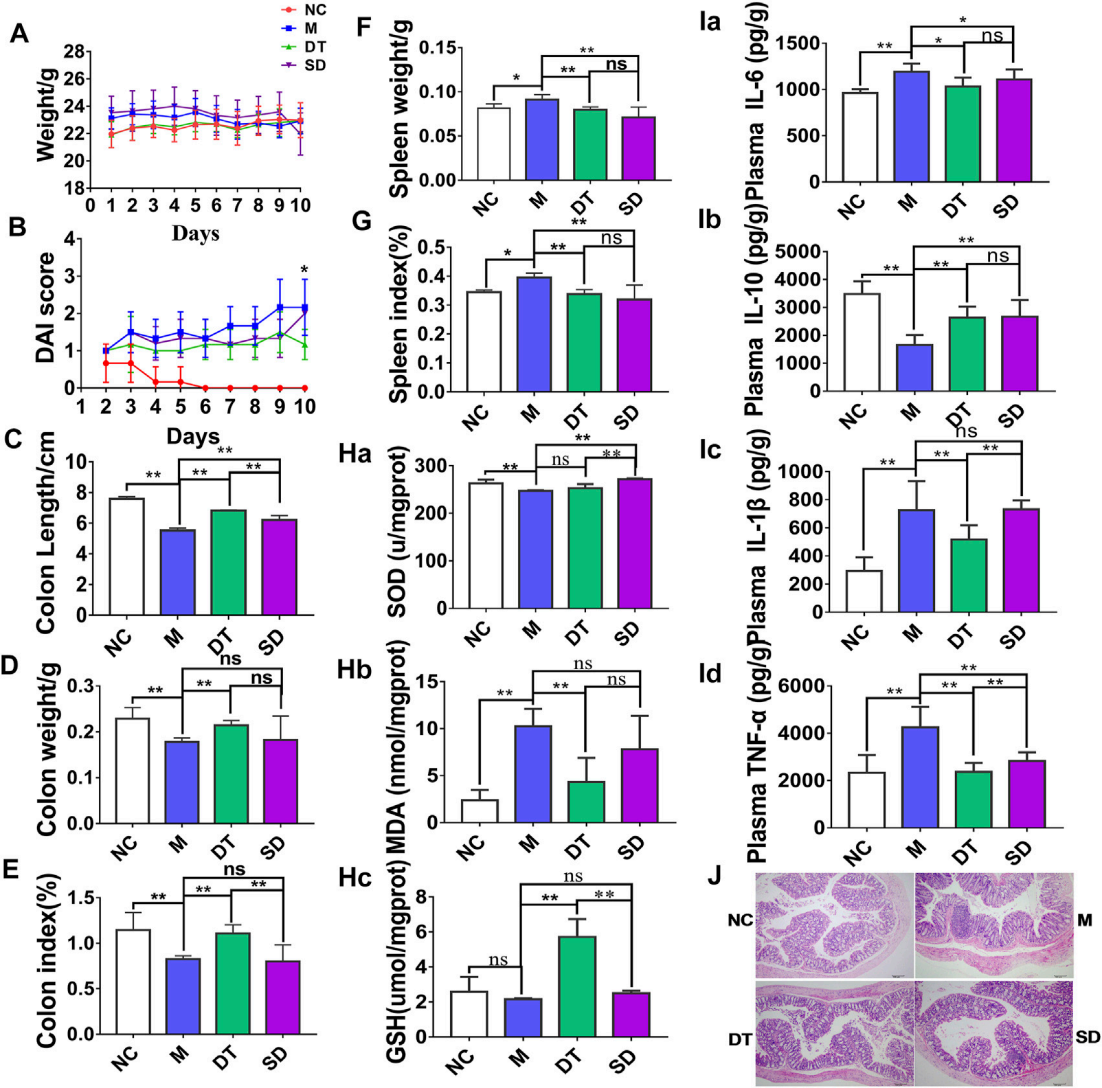
The curative effect of Scutellaria-Coptidis decoction and combined decoction on UC. **(A)** Body weight change. **(B)** DAI score. **(C)** Colon length. **(D)** Colon weight. **(E)** Colon index. **(F)** Spleen weight. **(G)** Spleen weight index. **(H)** SOD, MDA and GSH levels in colon tissue homogenate between NC, M, SD, and DT groups. **(I)** IL-6, IL-10, TNF-α, and IL-1β cytokines levels in colon tissue homogenate were measured by ELISA between NC, M, SD, and DT groups. **(J)** Representative microscopic pictures of H&E staining (100 x magnification). **(A–G)** *n* = 10 mice per group, **(H–J)** *n* = 6 mice per group, mean values ±SD are presented, *p < 0.05, **p < 0.01, ns not significant.

Scutellaria-Coptis decoction improved DSS-induced UC, and its effect on the DT group was more significant than that of the DSS group. Body weight loss (Figure 1A), shortening of colon length (Figures 1C–E), and spleen weight loss (Figures 1F, G) were significantly alleviated in the DT group when compared with SD group. In addition, DAI scores were consistent with the above results (Figure 1B) that shows an increasing trend. Colonic mucosal injury was evaluated by H&E staining. Mice in the M group had crypt loss, monocyte infiltration, and severe mucosal injury, while mice in the Scutellaria-Coptis treatment group exhibited reduced inflammatory cell infiltration, a relatively intact colonic structure, and less mucosal injury (Figure 1J).

The analyses results of colonic homogenates revealed that the contents of IL-1β, TNF-α, and IL-6 in colon tissues of mice in M group increased significantly (*p* < 0.01), while IL-10 content decreased significantly when compared with those of the NC group (*p* < 0.01, Figures 1Ia–Id), suggesting that there was a significant inflammatory response in mice in the M group. The contents of IL-1β, TNF-α, IL-6, and IL-10 in colon tissues of UC mice decreased significantly upon treatment with Scutellaria-Coptis decoction. A significant decrease was observed in the contents of IL-1β and TNF-α in DT group when compared with SD group (*p* < 0.01). The results suggest that the combined decoction of Scutellaria-Coptis exhibited a significant anti-inflammatory activity and could treat DSS-induced in UC mice by inhibiting inflammatory response. Moreover, the effect of DT was more significant than that of SD.

The anti-oxidation capacity of colon tissue homogenates of each group of mice was evaluated. The results showed that MDA content associated with the oxidative stress products in colon tissue homogenates of mice in the M group increased significantly after modeling (Figure 1Hb), whereas the levels of the antioxidant, GSH, and antioxidant enzyme, SOD activity reduced significantly (Figures 1Ha–Hc). A strong inflammatory response causes a certain degree of oxidative stress. Scutellaria-Coptis decoction increased GSH content and SOD activity, and reduced MDA content (*p* < 0.01). Furthermore, GSH content in the DT group increased significantly, while MDA content decreased significantly when compared with those of the SD group. The results also revealed that the curative effect of Scutellaria-Coptis decoction on alleviation of DSS-induced oxidative stress through antioxidative effects was significantly higher in the DT group that in the SD group.

Overall, the combined decoction of Scutellaria-Coptis significantly relieved UC in mice, with a significantly higher effect in the DT group than in the SD group. Therefore, the DT group was used in follow-up experiments.

### 3.2 Effect of Scutellaria-Coptis Decoction on UC is Determined by the Activities of Intestinal Flora

**FIGURE 2:**
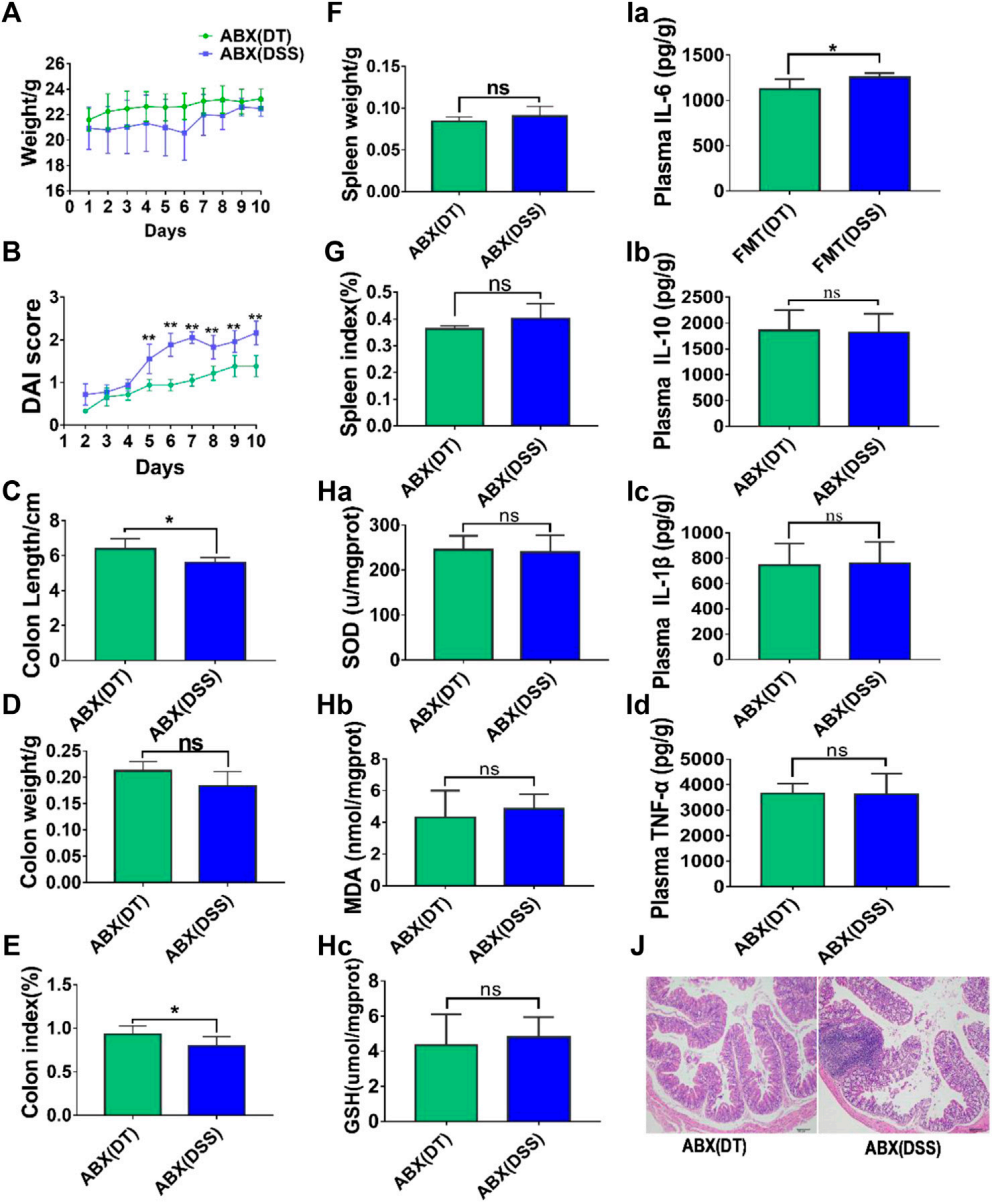
Scutellaria-Coptis anti UC depends on the action of intestinal microbe. **(A)** Body weight change. **(B)** DAI score. **(C)** Colon length. **(D)** Colon weight. **(E)** Colon index. **(F)** Spleen weight. **(G)** Spleen weight index. **(H)** SOD, MDA, and GSH levels in colon tissue homogenate between ABX (DT) and ABX (DSS) groups. **(I)** IL-6, IL-10, TNF-α, and IL-1β cytokines levels in colon tissue homogenate were measured by ELISA between ABX (DT) and ABX (DSS) groups. **(J)** Representative microscopic pictures of H&E staining (100x magnification). **(A–G)** *n* = 10 mice per group, **(H–J)** *n* = 6 mice per group, mean values ±SD are presented, *p < 0.05, **p < 0.01, ns not significant.

A combination of four different antibiotics was used to partially eliminate intestinal flora in mice before subjection to DSS-induced UC, and the ABX model was established. Notably, no significant differences were observed in body weight (Figure 2A), colon length (Figure 2C), colon weight (Figure 2D), DAI scores (Figure 2B), spleen weight (Figure 2F), spleen weight index (Figure 2G), and histomorphology (Figure 2J) between the ABX (DSS) and the ABX (DT) groups after intestinal microflora clearance. The levels of inflammatory and oxidative stress factors in colon tissues of pseudo-sterile mice were determined by ELISA. The results revealed that the levels of pro-inflammatory cytokines including IL-1β, TNF-α, IL-6, and anti-inflammatory cytokine, IL-10 between the ABX (DSS) and ABX (DT + DSS) groups were similar. Also, the levels of oxidative stress factors including SOD, MDA, and GSH between the ABX (DSS) and ABX (DT + DSS) groups were similar; no significant difference was observed between the two groups (Figures 2H, I). The effect of combined Scutellaria-Coptis decoction on UC, including alleviating symptoms, and inhibiting inflammatory response and antioxidant response, are closely associated with intestinal flora.

### 3.3 Fecal Microbial Transplantation Enhances the Capacity of Scutellaria-Coptis Decoction to Alleviate UC

**FIGURE 3:**
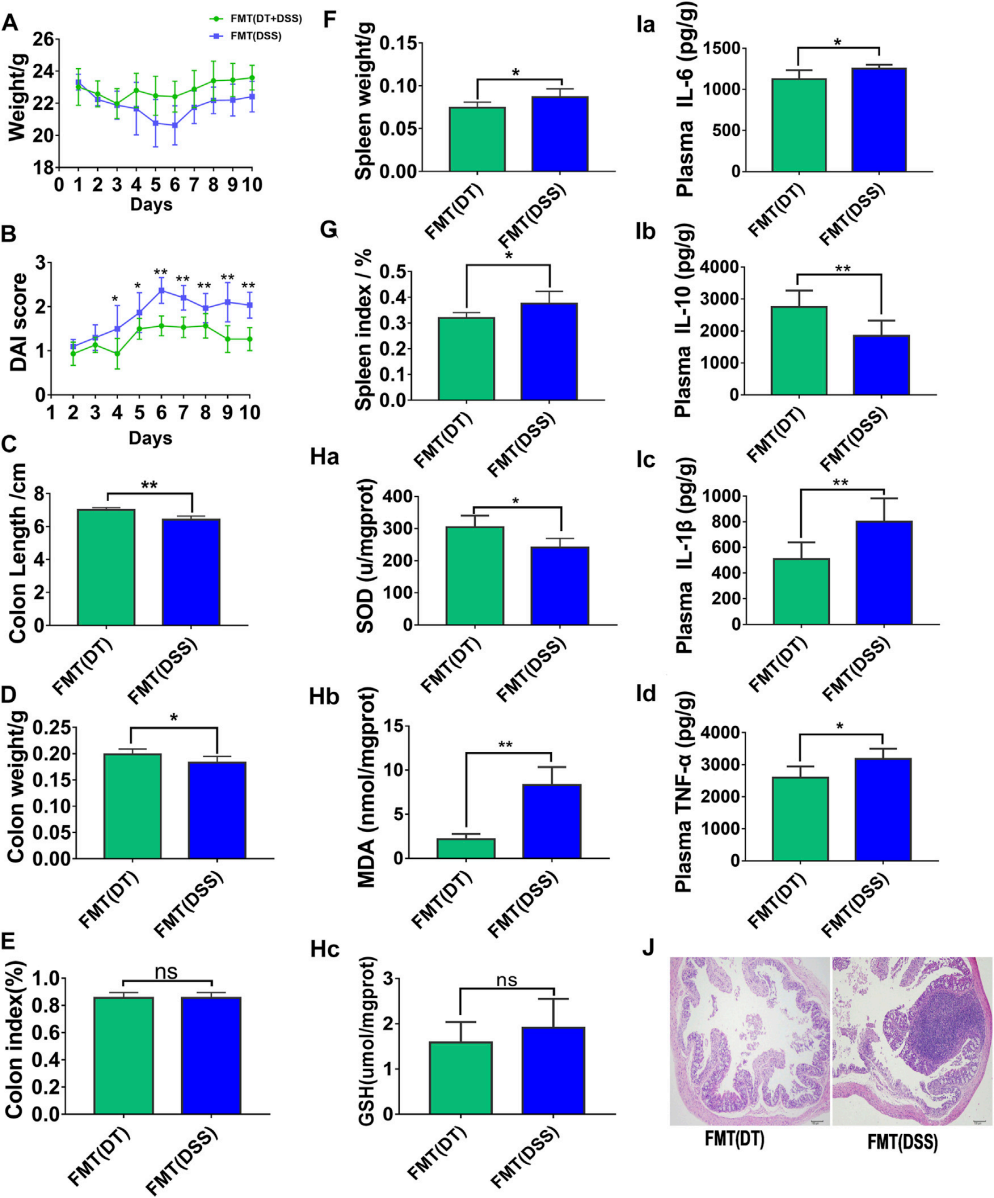
Fecal microbe transplantation can help Scutellaria-Coptis to resist UC. **(A)** Body weight change. **(B)** Disease activity index (DAI) score. **(C)** Colon length. **(D)** Colon weight. **(E)** Colon index. **(F)** Spleen weight. **(G)** Spleen weight index. **(H)** SOD, MDA, and GSH levels in colon tissue homogenate between FMT(DT) and FMT(DSS) groups. **(I)** IL-6, IL-10, TNF-α, and IL-1β cytokines levels in colon tissue homogenate were measured by ELISA between FMT (DT) and FMT (DSS) groups. **(J)** Representative microscopic pictures of H&E staining (100 x magnification). **(A–G)** *n* = 10 mice per group, **(H–J)** *n* = 6 mice per group, mice per group, mean values ±SD are presented, *p < 0.05, **p < 0.01, ns not significant.

To confirm whether the treatment of colitis in mice with combined Scutellaria-Coptis decoction depended on intestinal flora, the ABX model was established through intragastric administration of an antibiotic mixture to all experimental mice. The results revealed that colon inflammation in FMT (DT, GDWT) mice reduced significantly when compared with FMT (DSS, GDWT) group, which was reflected by changes in body weight (Figure 3A), DAI scores (Figure 3B), colon length (Figure 3C), colon weight (Figure 3D), colon index (Figure 3E), spleen weight (Figure 3F), spleen weight index (Figure 3G), inflammation factors associated with colon tissues (Figure 3I), oxidative stress levels (Figure 3H), and other results. Histomorphology results revealed that colon tissues of mice subjected to FMT (DT, GDWT group) exhibited a lower infiltration of inflammatory cells, relatively intact colon structure, and less mucosal damage when compared to the FMT (DSS, GDWT) group (Figure 3J). Moreover, the results confirmed that the combined decoction of Scutellaria-Coptis was closely associated with intestinal flora in mice.

### 3.4 Effect of Scutellaria-Coptis Decoction on Intestinal Microflora of Mice With UC

#### 3.4.1 Comparative Analysis of Abundance and Microbial Composition in Mice With UC

**FIGURE 4:**
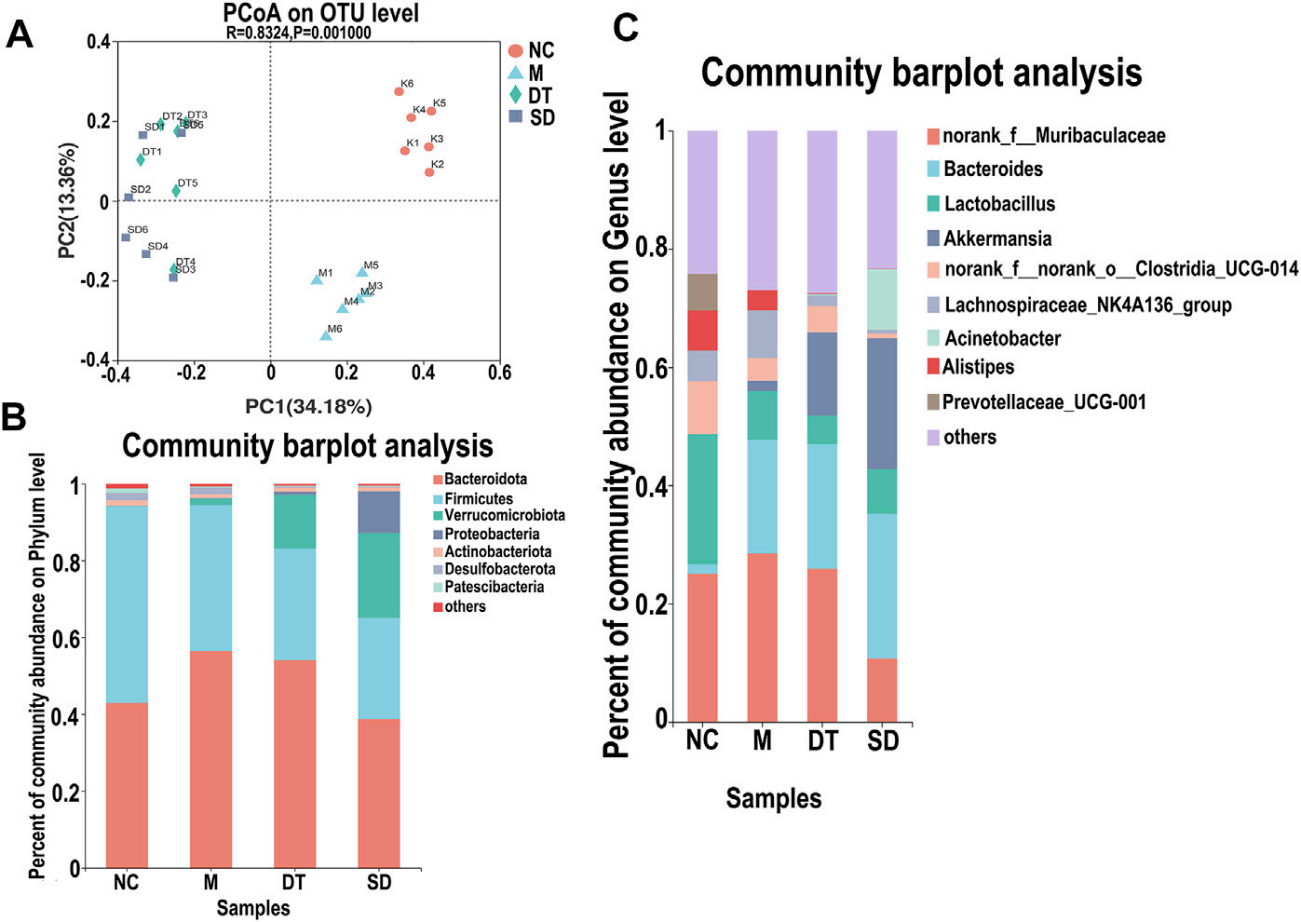
The relative abundance analysis of microbiome composition in ulcerative colitis-induced mice. **(A)** Principal component analysis of the gut microbiota. Each plot represents one sample. **(B)** Relative abundance of taxa at the phylum level. **(C)** Relative abundance of taxa at the genus level. **(A–C)** *n* = 10 mice per group.

**FIGURE 5:**
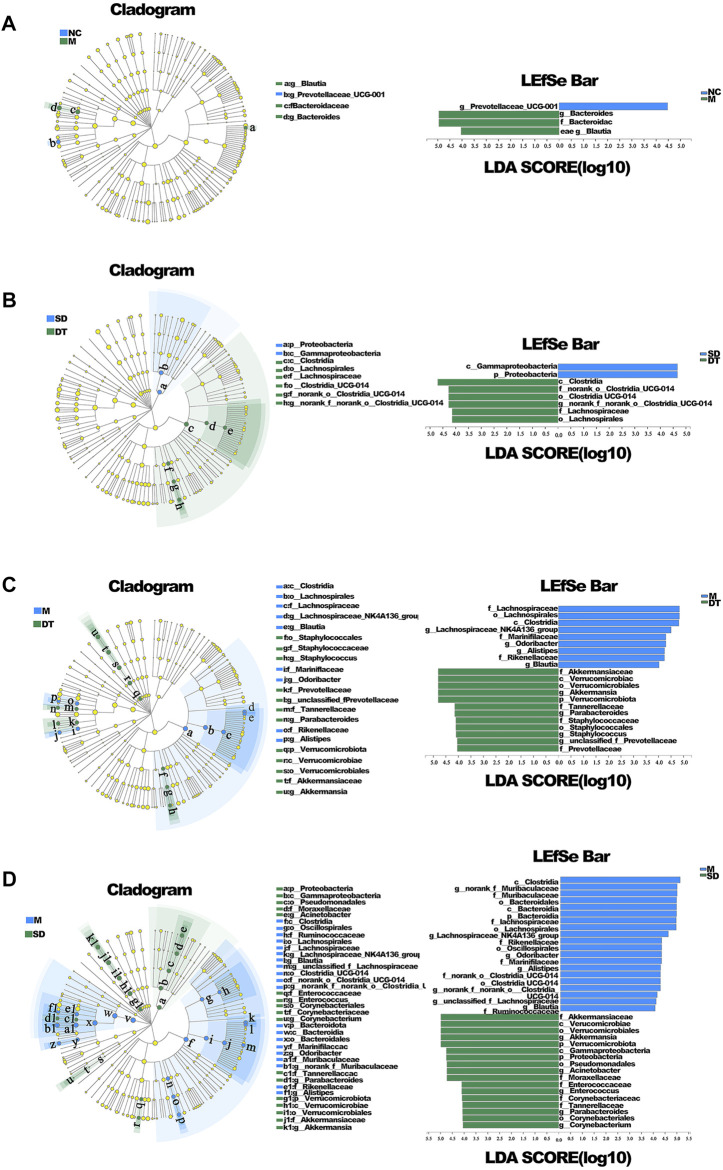
LEfSE analysis of microbiome composition in ulcerative colitis mice. **(A)** LEfSE analysis of NC and DSS group. **(B)** LEfSE analysis of SD and DT group. **(C)** LEfSE analysis of M and DT group. **(D)** LEfSE analysis of M and SD group. **(A–D)** *n* = 6 mice per group.

The variations in intestinal flora in UC mice treated with Scutellaria-Coptis decoction were analyzed by high-throughput sequencing. The results of principal component analysis revealed that the composition of intestinal flora of mice in the M group was significantly different from that of the NC group (Figure 4A). The intestinal flora of the DT and SD groups differed from those of the M group. The distance between the SD and M groups was closer than that between the DT and M groups. The microbial flora communities of all the groups at the phylum level were similar; Bacteroidetes and Firmicutes were the dominant phyla. The relative abundance of Bacteroidetes in M, SD, and DT groups increased significantly, while the relative abundance of Firmicutes decreased significantly when compared to those of the NC group. However, the abundance of Firmicutes decreased gradually in the DT group when compared with that of the SD group (Figure 4B). Based on the genus level, *norank_f__Muribaculaceae*, *Bacteroides*, lactic acid bacteria, and *Akkermansia* were the dominant genera. The abundances of *Norank-F-Muribaculaceae* and *Akkermansia* were restored in SD and DT groups. Notably, the abundance of *Norank-F-Muribaculaceae* in the DT group was almost similar to that of the NC group (Figure 4C). Linear discriminant analysis effect size (LEfSe) analysis revealed similar results (Figures 5A–D).

#### 3.4.2 Differences in Species Compositions

**FIGURE 6:**
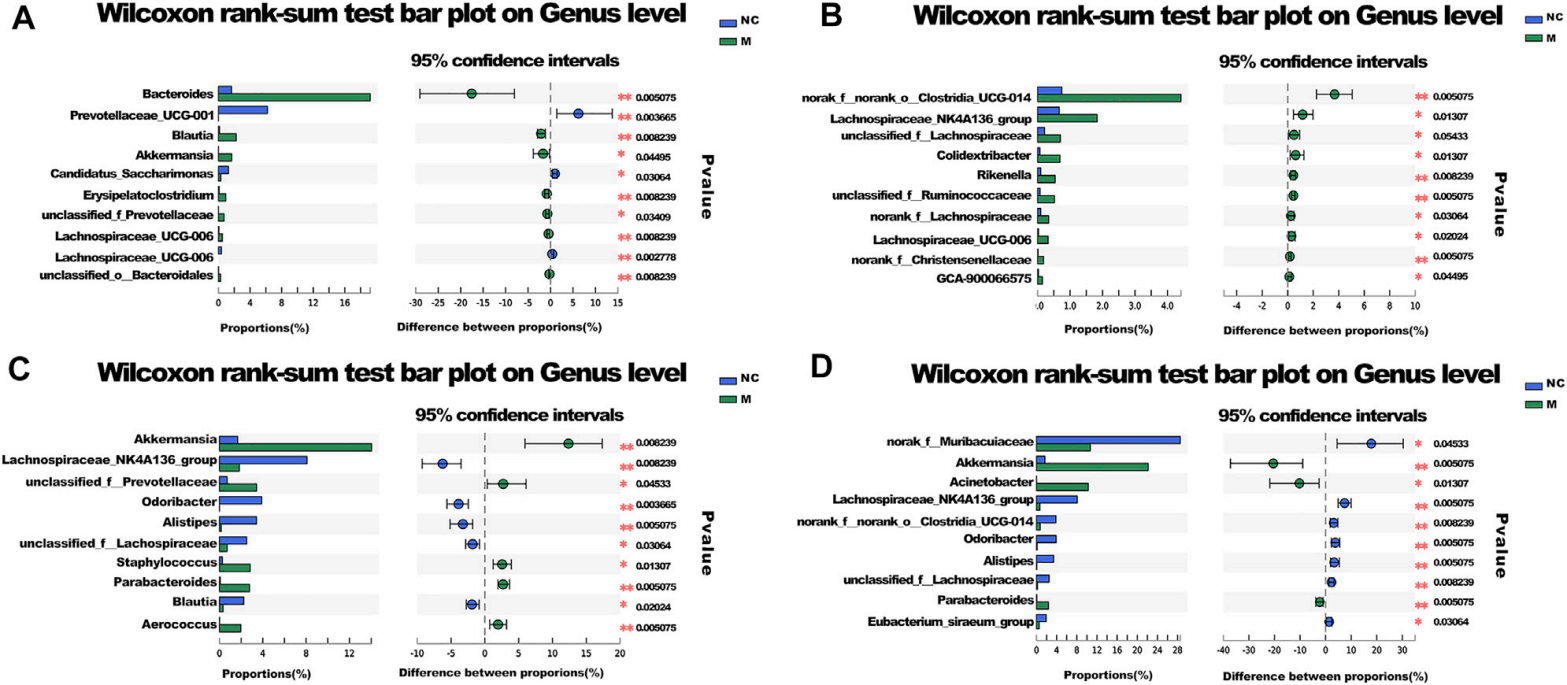
Species difference analysis. **(A)** The different species between NC and M group. **(B)** The different species between SD and DT group. **(C)** The different species between M and DT group. **(D)** The different species between M and SD group. **(A–D)** *n* = 6 mice per group, mean values ±SD are presented, *p < 0.05, **p < 0.01.

To determine the abundances of various species in different groups of microbial communities based on microbial community abundance data for the samples, the significant differences in communities between the groups were evaluated at the genus level (Figure 6).

The abundances of *Bacteroides* and *Blautia* in the M group increased significantly, while the abundance of *Prevotellaceae UCG-001* decreased considerably when compared with those of the NC group. The abundances of *Akkermansia* and *Parabacteroides* increased significantly in SD and DT groups, while the abundances of *Odoribacter* and *Alistipes* decreased when compared with those of the M group. Moreover, the abundance of *norank_f__Muribaculaceae* in the DT group increased relatively. The abundances of *Bacteroides*, *Akkermansia*, and *Lactobacillus* also changed, although the difference was not significant. The results suggest that *Bacteroides* was the key species of intestinal flora in DSS group, whereas *Akkermansia* could influence the treatment of DSS-induced colitis with Scutellaria-Coptis decoction (Chen et al., 2017; Liu et al., 2021).

### 3.5 Scutellaria-Coptis Decoction Alleviates the Correlation Between Intestinal Microflora and Inflammatory Factors in UC Mice

**FIGURE 7:**
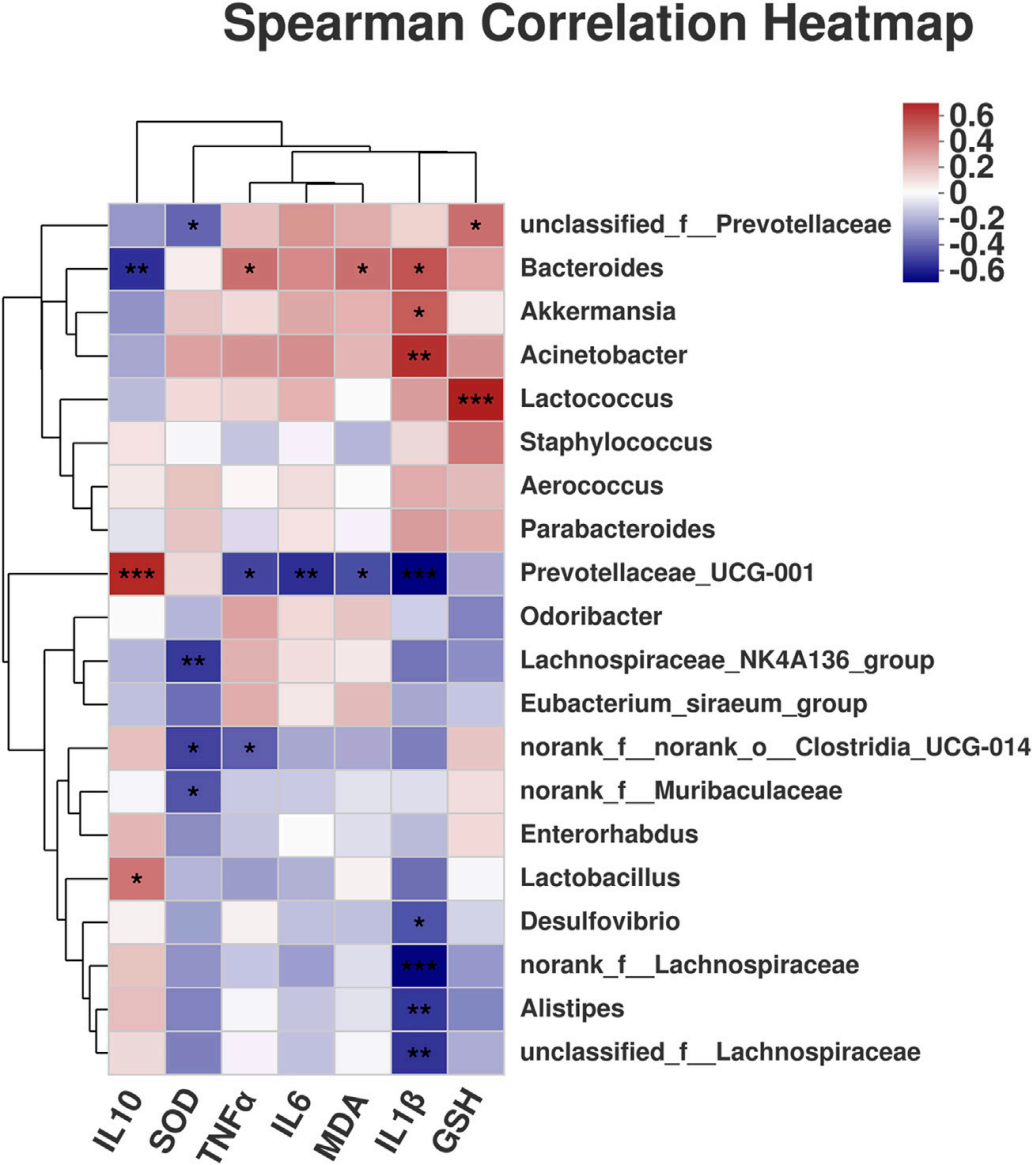
The correlation between intestinal microflora and inflammatory factors.

A correlation heatmap was used to visualize the relationships between different species in a sample and clinical variables through correlation values, and to evaluate the correlation between microbial classification and clinical variables. *Prevotellaceae UCG-001* was significantly positively correlated with IL-10 but significantly negatively correlated with IL-1β, IL-6, TNF-α, and MDA (Figure 7). *Bacteroides* exhibited a significant positive correlation with IL-1β, TNF-α, and MDA but a significant negative correlation with IL-10. *Akkermansia* was positively correlated with IL-1β, *Acinetobacter* was positively correlated with IL-1β, and *Lactococcus* was positively correlated with GSH. The results suggest that the inflammatory factors were closely associated with intestinal microflora in the alleviation of UC. Therefore, the alleviation of UC by Scutellaria-Coptis decoction is the result of the co-regulation of inflammatory factors and intestinal microflora. The results further verified that the treatment of UC by Scutellaria-Coptis is dependent on intestinal flora.

## 4 Discussion

**FIGURE 8:**
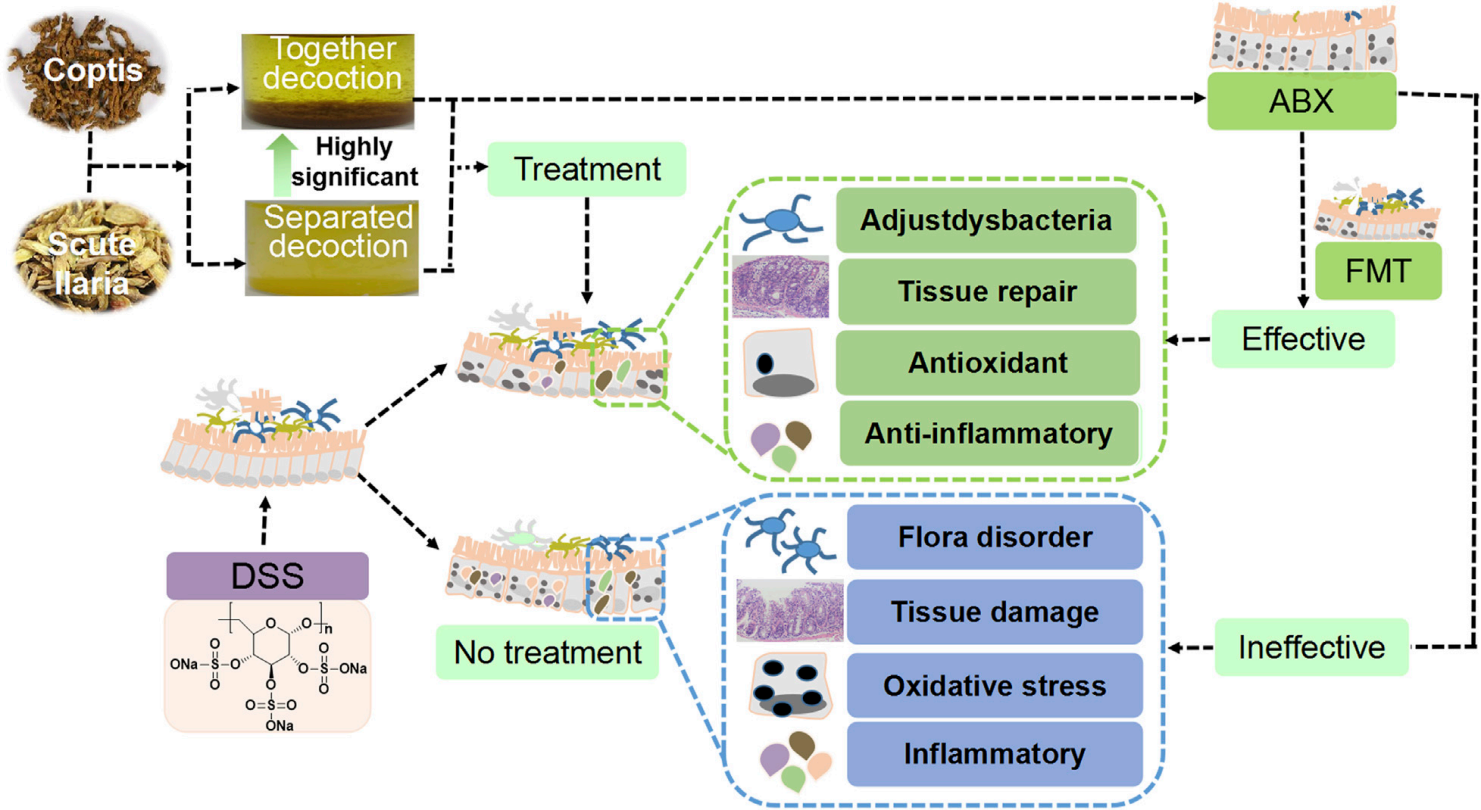
Scutellaria-Coptis can relieve UC and the effect is highly significant when a combined decoction is used, which can alleviate colon inflammation depending on intestinal microbes to a certain extent.

The effects of separated and combined Scutellaria-Coptis decoction on UC were investigated in the present study. The results revealed that the effect of combined decoction of Scutellaria-Coptis on DSS-induced UC was stronger than that of the separate decoction. The effects of Scutellaria-Coptis were reflected by weight loss, low DAI scores, shortening of the colon length, histological grading, substantial variations in the levels of inflammatory factors, and enhancement of antioxidant capacity (Beaudry et al., 2019; Daskalaki et al., 2019; Liu et al., 2020). The diversity and composition of intestinal flora were rich, which was conducive to the production of anti-inflammatory factors and changes in antioxidant stress levels of colon tissues, in turn, alleviating UC symptoms after treatment of UC mice with combined decoction of Scutellaria-Coptis (Liu et al., 2020). Furthermore, ABX and FMT models confirmed that Scutellaria-Coptis alleviated UC in mice based on the composition of the gut microbiota (Liu et al., 2020; Benech and Sokol, 2020; Liu et al., 2021; Draper et al., 2018). Therefore, we inferred that the traditional method of extracting Scutellaria-Coptis decoction exerts a superior effect to that of separate decoction, which mainly treats UC by regulating intestinal flora, inflammation, and oxidative stress (Figure 8).

Reduced diversity and composition of intestinal microflora in patients with UC is considered a disorder, which is characterized by the loss of beneficial symbiotic flora and the increase in pathogenic bacteria (Wang et al., 2019; Yang et al., 2021). 16S rRNA sequencing was used to study the changes in microbial diversity and composition after Scutellaria-Coptis treatment. Treatment with Scutellaria-Coptis decoction significantly increased the diversity of intestinal microflora and altered the microbial community structure when compared with DSS treatment group. Previous studies have reported that *Blautia* is a key flora associated with UC (Gough et al., 2015; Lan et al., 2017; Wei et al., 2020). Notably, two typical bacterial groups, including *Akkermansia* and *Lactobacillus*, which facilitate the synthesis of intestinal microbial metabolites, such as short chain fatty acids, were relatively enriched in SD and DT groups (Liu et al., 2021). In addition, *Akkermansia* as a novel anticancer “star bacterium”, provides nutrients to the intestinal mucosa, promotes intestinal barrier function, generates certain molecular signals, stimulates and strengthens the intestinal wall, in addition to exhibiting anti-cancer, anti-aging, and anti-diabetic effects. *Lactobacillus* protects the intestinal mucosal barrier by antagonizing pathogenic bacteria mediated by its endogenous defense barrier function, promotes secretion of mucin and secretory IgA in intestinal epithelial cells, and repairs the intestinal mucosal barrier (Endesfelder et al., 2016; Borton et al., 2017). Therefore, administration of separated and combined Scutellaria-Coptis decoction can adjust the intestinal flora imbalances caused by DSS-induced UC by increasing the biological diversity of intestinal flora and promoting the relative abundances of beneficial bacteria.

The use of compounded medicine is a clinical practice of traditional Chinese medicine, which is based on the principle that “a single component exerts an insignificant effect, whereas several components exert a significant effect.” Similarly, the chemical composition of compound drugs of the traditional Chinese medicine after the co-decoction of multiple drugs is not a simple addition of the chemical components of a single medicine in the prescription. There are several interactions occurring among the multiple components, such as solubilization, dissolution promotion, inhibition of dissolution, and various chemical reactions, which are beneficial or reduce their clinical efficacy **(** Wei et al., 2020 **)**. The two different extraction methods applied in combined and separated decoctions of Scutellaria-Coptis significantly influenced the contents of key active components in the extract. The contents of berberine hydrochloride and baicalin in the combined decoction decreased by 40 and 35%, respectively, when compared with those of the separated decoction of Scutellaria-Coptis. The observations could be explained as follows. First, baicalin and berberine hydrochloride underwent an acid-base neutralization reaction under the hot and humid conditions of decocting Scutellaria-Coptis, forming numerous water-soluble flocculent complexes and precipitates. Second, the complex adsorbed a large amount of baicalin and berberine hydrochloride, which significantly reduced the contents of the two components in the decoction. Previous studies have reported that the complex also alleviates UC. Strikingly, the results of the present study revealed that the contents of key active ingredients were significantly lower in the separated decoction than in the combined decoction, which exerted a stronger anti-UC effect than the separate decoction. That is, the complex not only destroys the active ingredients but it also exhibits medicinal effects.

Traditional Chinese medicines are composed of several precipitated formulas, such as rhubarb, three yellow, aconite, and licorice formulas. Banxia xiexin decoction in Zhongjing prescriptions, Baitouweng decoction, Gegen Qinlian decoction, Sini decoction, as well as recent prescriptions of Sanhuang gypsum decoction, Nei Shu Huang Lian decoction, and Peony-Glycyrrhiza decoction among others. Such prescriptions are easy to precipitate due to the compatibility of tannins and alkaloid glycosides, organic alkaloids, acids alkaloids, and other types of traditional Chinese medicines. The compounds are often removed in industrial processes. In modern industry, precipitation-compatible traditional Chinese medicines are treated separately to avoid the formation of precipitates, which is the conflict between modern medicine and traditional medicine practices, which are represented by Scutellaria-Coptis. The scientific connotation of traditional precipitant compatibility from the aspect of anti-UC efficacy has been studied, and the scientific requirements of simultaneous administration of self-precipitants and supernatants in traditional Chinese medicine decoctions verified. Notably, the effect of the combined decoction was stronger than that of separated decoction in the present study. Conversely, a strong positive correlation was not observed between the efficacy of traditional Chinese medicine and the contents of the main active components. Nevertheless, there are numerous forms of effective components of Traditional Chinese medicine, and self-precipitation in precipitate compatibility could be the “drug storage warehouse” of effective components (Luo et al., 2021). After entering the body, the precipitates are intercepted and absorbed by intestinal villi, and become active components or precursors again under the action of intestinal microorganisms and enzymes, thereby acting on intestines, microorganisms, and diffusing into the blood. Self-precipitation may retain the active ingredients to a certain extent for relatively long durations of efficacy than real solutions that rapidly pass through the gastrointestinal tract. Further research is required to validate the findings of the present study.

## Data Availability

The datasets presented in this study can be found in online repositories. The names of the repository/repositories and accession number(s) can be found below: National Center for Biotechnology Information (NCBI) BioProject database under accession number PRJNA810978.
